# Detection of *Haemophilus influenzae* by loop-mediated isothermal amplification coupled with nanoparticle-based lateral flow biosensor assay

**DOI:** 10.1186/s12866-022-02547-5

**Published:** 2022-05-05

**Authors:** Qilong Cao, Shaoshuai Liang, Feng Lin, Jun Cao, Lin Wang, Hui Li, Mengyang Liu, Yajuan Wang, Lijun Zhao, Xiaolong Cao, Yan Guo

**Affiliations:** 1 Biomedical Informatics & Genomics Center, Key Laboratory of Biomedical Information Engineering of Ministry of Education, School of Life Science and Technology, Xi’an Jiaotong University, Xi’an, Shaanxi, 710049 China; 2 Qingdao Haier Biotech Co. Ltd, Qingdao, 266109 China; 3 School of Medicine, Huaqiao University, Quanzhou, 362021 Fujian China; 4 Hefei National Laboratory for Physical Sciences at the Microscale, School of Life Sciences, Division of Life Sciences and Medicine, University of Science and Technology of China, Hefei, 230026 China; 5 Department of Clinical Laboratory, The Affiliated Hospital of Qingdao University, 59 Haier Road, Qingdao, 266035 Shandong China; 6 Dingzhou City People’s Hospital, Heibei, 073099 China; 7 Beijing Changping Institute for Tuberculosis Prevention and Treatment, Beijing, 102200 China

**Keywords:** *Haemophilus influenzae*, LAMP-LFB, Nanoparticle-based biosensor, Effective diagnostic approach

## Abstract

**Background:**

*Haemophilus influenzae* was the most aggressive pathogen and formed a major cause of bacterial meningitis and pneumonia in young children and infants, which need medical emergency requiring immediate diagnosis and treatment. However, From isolation to identification of *H. influenzae*, the traditional diagnose strategy was time-consuming and expensive. Therefore, the establishment of a convenient, highly sensitive, and stable detection system is urgent and critical.

**Results:**

In this study, we used a combined method to detect *H. influenzae*. Six specific primers were designed on the basis of outer membrane protein *P6* gene sequence of *H. influenzae*. The reaction condition such as the optimum temperature was 65℃, and the optimum reaction time was 30 min, respectively. Through the loop-mediated isothermal amplification (LAMP) in combination with nanoparticle-based lateral flow biosensor (LFB), the sensitivity of LAMP-LFB showed 100 fg was the lowest genomic DNA templates concentration in the pure cultures. Meanwhile, the specificity of *H. influenzae*-LAMP-LFB assay showed the exclusive positive results, which were detected in *H. influenzae* templates. In 55 clinical sputum samples, 22 samples were positive with LAMP-LFB method, which was in accordance with the traditional culture and Polymerase Chain Reaction (PCR) method. The accuracy in diagnosing *H. influenzae* with LAMP-LFB could reach 100%, compared to culture and PCR method, indicating the LAMP-LFB had more advantages in target pathogen detection.

**Conclusions:**

Taken together, LAMP-LFB could be used as an effective diagnostic approach for *H*. *influenzae* in the conditions of basic and clinical labs, which would allow clinicians to make better informed decisions regarding patient treatment without delay.

**Supplementary Information:**

The online version contains supplementary material available at 10.1186/s12866-022-02547-5.

## Background

*Haemophilus influenzae* is a common human pathogenic strain, which is related to a variety of serious childhood illnesses, such as pneumonia, otitis media, bacteremia and meningitis [1]. Previously, it is difficult to distinguish *H. influenzae* from *Haemophilus spp.*, such as *Haemophilus parainfluenzae*. The traditional detection techniques for isolation and identification of *H. influenzae,* involving colonial morphology, serological identification and growth assays, are usually consuming a lot of time and cumbersome [1]. Therefore, the establishment of a convenient, highly sensitive, and stable detection system is urgent and critical for early diagnosis and effective antibiotic therapy.

For clinical detection and analysis, the molecular detection technology has the value of sensitivity and specificity [2–5]. With different targeting genes, such as the outer membrane protein (OMP) *P6*, capsulation-associated protein Bex A [6], and rRNA-encoding genes [7, 8], the Polymerase Chain Reaction (PCR) method has been successfully applied to *H. influenzae* detection. Thereinto, OMP *P6* is a highly conserved gene in *H. influenzae* [9, 10]. Morever, it has became a potential vaccine component that protects against *H. influenzae* [11]. Hence, it is a suitable target gene for *H. influenzae* identification than other genes.

A detection technique of nucleic acid called loop-mediated isothermal amplification (LAMP) was established in 2000 [12]. The principle of LAMP is to design 3 pairs of specific primers based on the 6 regions of the 3' and 5' ends of the target gene, including 1 external primer, 1 ring primer and 1 internal primer. The 3 pairs of specific primers rely on the chain replacement *Bst* DNA polymerase, an appropriate temperature range from 60℃ to 67℃ to make the chain replacement DNA synthesis self-cycle continuously, so as to achieve rapid amplification. After the reaction, the amplification can be judged by the turbidity of the precipitation of magnesium pyrophosphate, the by-product of the amplification, or the fluorescent dye. In this reaction, dumbbell-shaped template was formed first, and then cyclic amplification was carried out, followed by elongation and cyclic amplification [12–14]. For LAMP method, it possesses many advantages, including high sensitivity and specificity, regulable pH and the temperature ranges for amplification, and shorter time consumption (less than one hour) [15, 16]. In addition, the reagents used in LAMP assay are not expensive [15, 16]. Many studies published previously, used the LAMP method to detect bacteria, viruses, and parasites [17, 18]. For example, *H. influenzae*, *H. influenzae* type b (Hib), and serotype of non-Hib have been detection with LAMP method to diagnose for patients [1, 19, 20]. More recently, numerous methods involving electrophoresis, turbidimeters, nanoparticle-based lateral flow biosensors (LFBs) and color agents have been used to analyze the amplification production of LAMP [21]. In brief, the LFB including the sample pad, membrane backing card, nitrocellulose membrane (NC), conjugate pad, and absorbent pad, which were assembled onto a plastic adhesive backing card. In the view of low cost, simplicity and rapidness, various LFB are derived out and widely used to analyze LAMP amplicon [22–25]. It is worth mentioning that the multiple reverse transcription LAMP combined with LFB have been used to diagnosis coronavirus disease 19 (COVID-19) [26]. For the above favorable characteristics, we used LAMP combined with LFB assay and OMP *P6* gene to specifically detect *H. influenzae*. With strain pure cultures and clinical samples, the optimal reaction conditions, sensitivity, specificity and feasibility of *H. influenzae* detection strategy were validated.

## Results

### Confirmation and demonstration of LAMP products

**Fig. 1:**
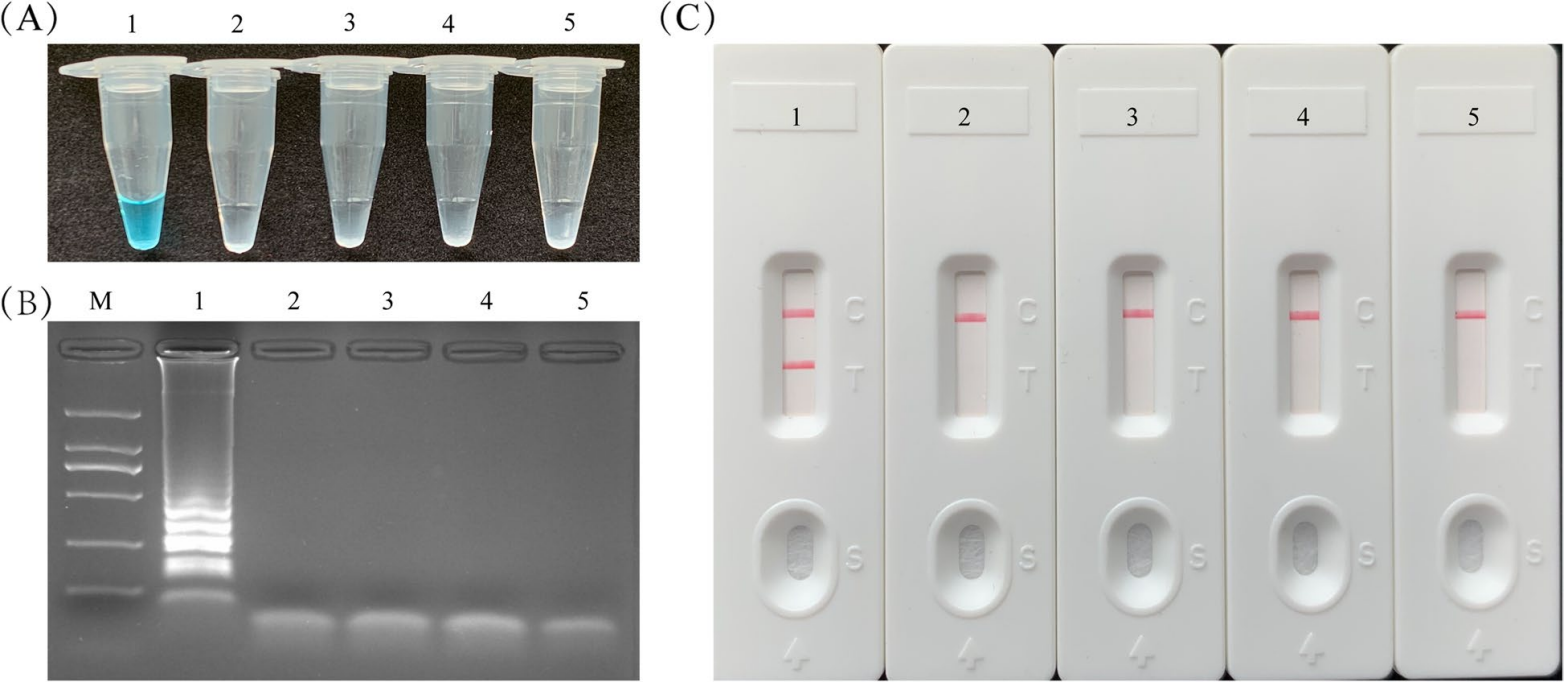
Confirmation and demonstration of *H. influenzae*-LAMP amplification products. **A** The amplification products’ color change of *H. influenzae*-LAMP assay was showed with colorimetric indicators; **B** Amplicon of *H. influenzae*-LAMP was analyzed by agarose gel electrophoresis; **C** The products of *H. influenzae*-LAMP were detected with LFB. Tube 1/lane 1/biosensor 1, positive amplification of *H. influenzae*; tube 2/ lane 2/ biosensor 2, negative control of *H. parainfluenzae*; tube 3/ lane 3/ biosensor 3, negative control of *H. haemolyticus*; tube 4/ lane 4/ biosensor 4, negative control of *S. aureus*; tube 5/ lane 5/ biosensor 5, black control (double distilled water, DW)

For the purpose of assessing the effectiveness of *H. influenzae* LAMP primers, the LAMP reactions were conducted with *H. influenzae*, *H. parainfluenzae*, *H. haemolyticus*, and *non-H. influenzae* genomic templates at 63℃ for 1 h. After adding the VDR reagents into the amplification mixtures, the color of positive amplification products of LAMP in tube changed from colourless to light blue (Fig. 1A). Meanwhile, in the negative control and blank tube, the color remained colorlessness. The LAMP amplification products were detected by 2% agarose gel electrophoresis, which presented the ladder bands only in the positive reaction, and no bands in the blank and negative control **(**Fig. 1B). With LFB, in the positive amplifications, the clear visible two red bands were seen for the control line (CL) and test line (TL). While, in the negative and blank controls, only a red band CL was observed (Fig. 1C). These results confirmed that the primers were suitable for *H. influenzae* detection with LAMP-LFB assay.

### Optimal amplification temperature of *H. influenzae* LAMP primers

**Fig. 2:**
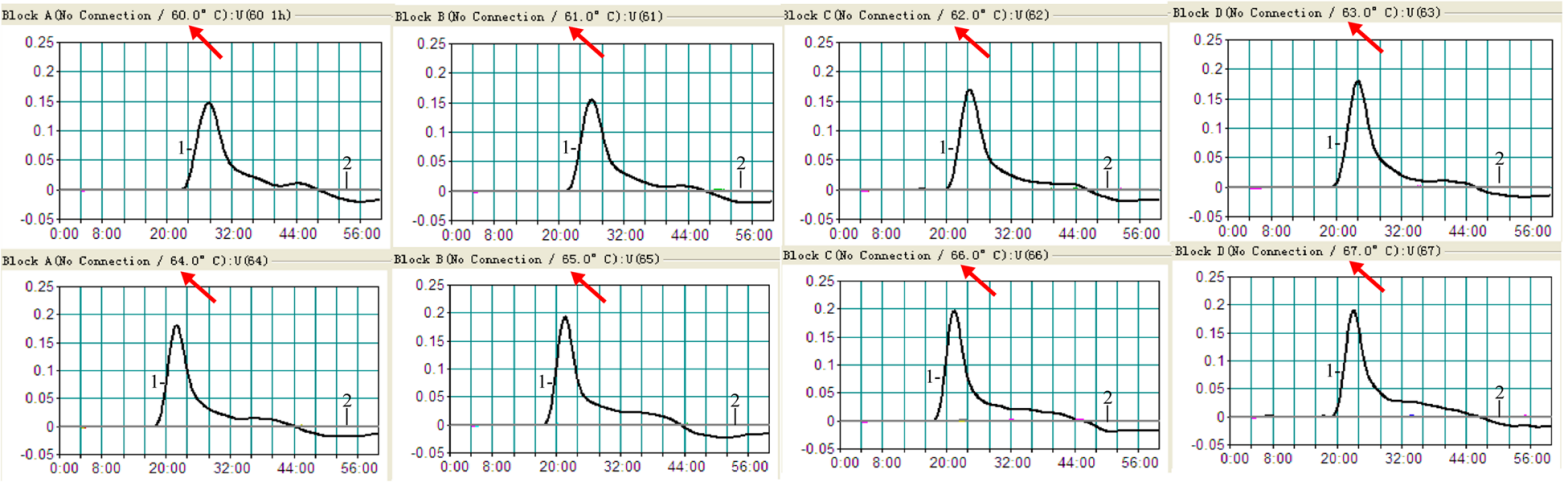
Optimization of amplification temperature for *H. influenzae*-LAMP assay. The LAMP method for *H. influenzae* detection was monitored with real-time turbidimeter. Eight temperatures and their corresponding curves were showed in the pictures. The turbidity > 0.1 was considered as the positive result (threshold value was 0.1). The *H. influenzae* DNA templates used in each reaction was 1 pg. Eight pictures (**A**-**H**) were acquired from a temperature range with 1℃ intervals (from 60℃ to 67℃). Signal 1, positive amplification of *H. influenzae*; signal 2, negative control of *S. aureus*

To test the optimum reaction temperature of *H. influenzae* LAMP primers, the LAMP amplifications were conducted with genomic DNA of *H. influenzae* of 10 pg/μl per reaction from 60℃ to 67℃ with 1℃ interval. Eight motorial graphs matching with corresponding temperature were acquired by detecting the amplification products with real-time turbidimeter. The amplification of OMP *P6* gene could be found at all tested temperatures. However, the 65℃ was the optimal temperature, because the absorbance threshold was reached first (Fig. 2).

### Analytical sensitivity of *H. influenzae* LAMP-LFB detection

**Fig. 3:**
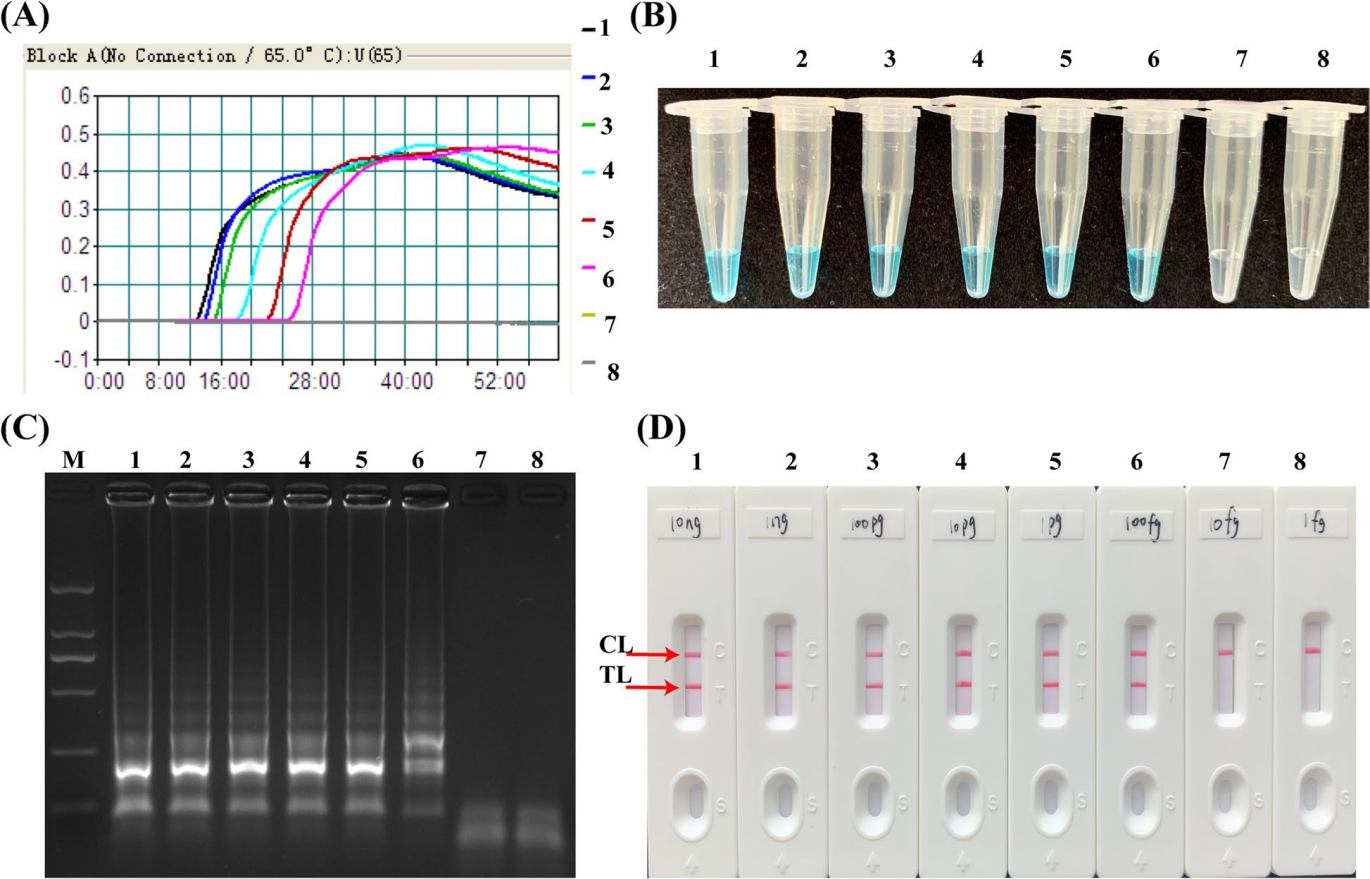
Sensitivity of *H. influenzae* LAMP-LFB assay with gradient dilution of genomic DNA templates. Four monitoring method: **A** Real-time turbidimeter; **B** Colorimetric Indicator; **C** Agarose gel electrophoresis; **D** Biosensors. 1–8 represented the DNA template of each reaction was 10 ng, 1 ng, 100 pg, 10 pg, 1 pg, 100 fg, 10 fg, 1 fg, respectively. The genomic DNA from 10 ng to 100 fg produced positive result

The genomic DNA of *H. influenzae* was diluted into a series of gradients (10 ng, 1 ng, 100 pg, 10 pg, 1 pg, 100 fg, 10 fg, and 1 fg per mixture), which were used as the templates to analyze the sensitivity of LAMP-LFB assay. As was shown in Fig. 3, the limit of detection (LOD) of LAMP-LFB assays was 100 fg by the four detectiong methods. These results indicated that LAMP detection’s LOD of *H. influenzae* by real-time turbidimeter (Fig. 3A), colorimetric indicator (Fig. 3B), and 2% agarose gel electrophoresis (Fig. 3C) were conformity with LFB analysis (Fig. 3D).

### The optimal duration time of *H. influenzae* LAMP-LFB assay

**Fig. 4:**
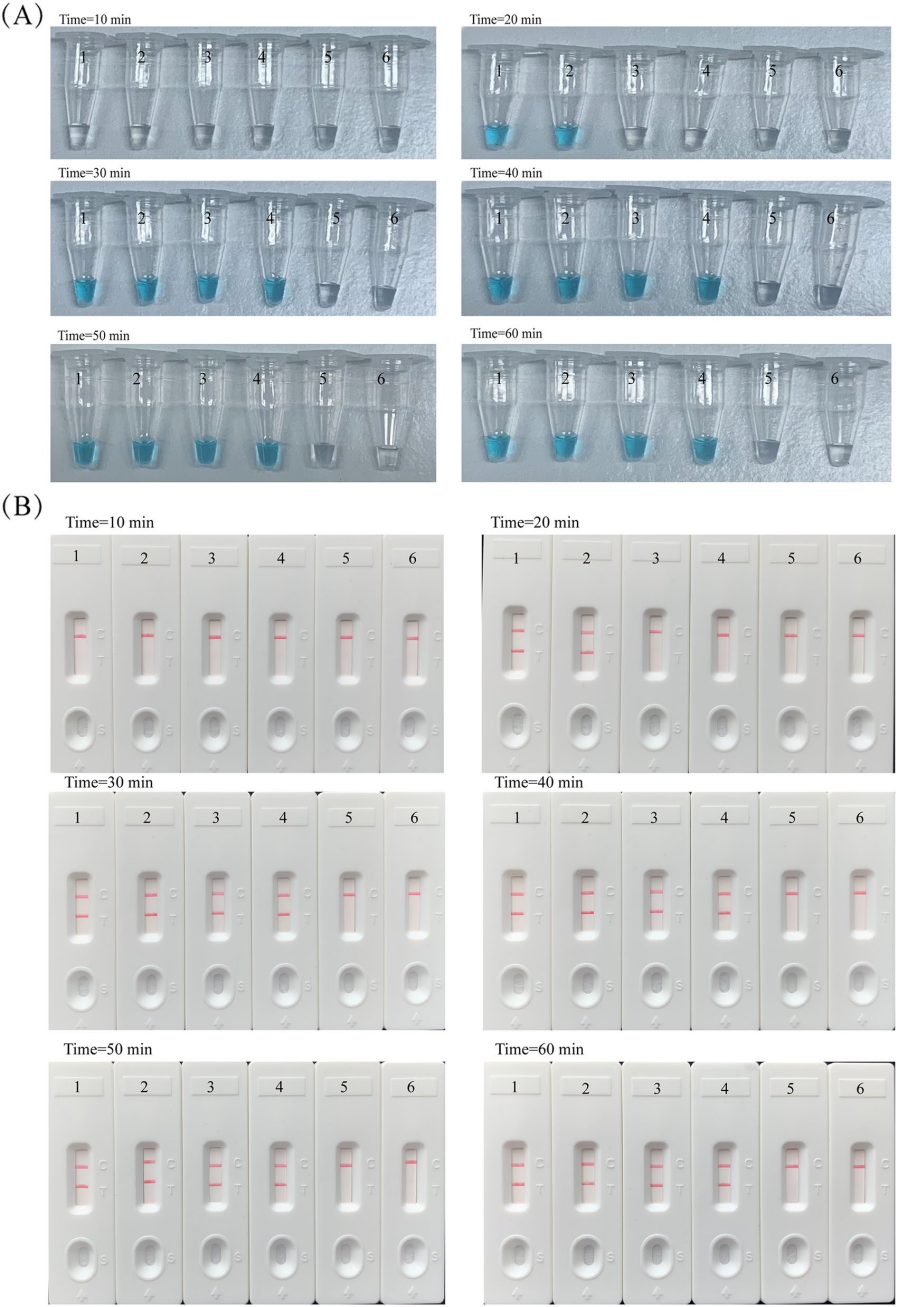
Optimal duration time of *H. influenzae-*LAMP-LFB assay. **A** Colorimetric Indicator; **B** LFB. Six reaction times: 10 min; 20 min; 30 min; 40 min; 50 min; 60 min were measured at 65℃. The 1–6 biosensors represented different genomic DNA template levels of *H. influenzae,* including 100 pg, 10 pg, 1 pg, 100 fg, 10 fg, and blank control (DW), respectively. The optimal time was 30 min

In order to examine the optimal duration time of *H. influenzae* LAMP-LFB assay, the assay were conducted at 65℃. And the time range was set from 10 to 60 min with 10 min interval. With 100 fg genomic DNA of LOD level, the sufficient time for LAMP assay with colorimetric indicators and LFB were both only 30 min (Fig. 4A and B). Take together, the whole detection process of *H. influenzae* LAMP-LFB, involving genomic DNA preparation of 20 min, LAMP reaction of 30 min, and LFB analysis of 2 min, took only 52 min.

### Specificity of *H. influenzae* LAMP-LFB assay

**Fig. 5:**
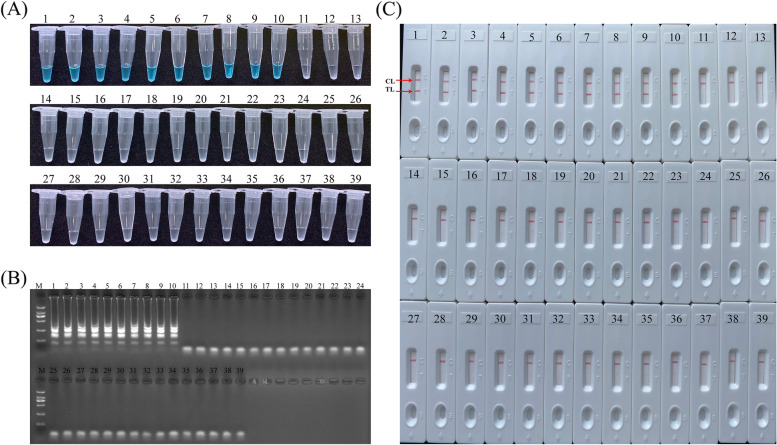
Specificity of *H. influenzae* LAMP-LFB assay with different strains’ DNA templates. **A** The visible color change of *H. influenzae*-LAMP amplicon was observed with colorimetric indicators; **B** The LAMP products were detected with agarose gel electrophoresis; **C** The LAMP assay were analyzed by means of later flow biosensor. 1–10, *H. influenzae*; 11–13, *H. parainfluenzae*; 14–16, *H. haemolyticus*; 17–19, *H. parahaemolyticus*; 20–22, *Kleber pneumoniae*; 23–24, *Pseudomonas aeruginosa*; 25–26, *Citrobacter braakii*; 27, *Streptococcus agalactiae*; 28, *Staphylococcus haemophilus*; 29, *Proteus mirabilis*; 30–31, *Streptococcus sui*s; 32–33, *Listeria monocytogenes*; 34, *Listeria innocua*; 35–39, *Escherichia coli*

In this study, the specificity of the LAMP-LFB method was evaluated with the genomic templates extracted from 10 *H. influenzae* strains, 3 *H. parainfluenzae*, 3 *H. haemolyticus*, 3 *H. parahaemolyticus* and 20 non-*H. influenzae* bacterial pathogens (Table 2). As shown in Fig. 5, with three methods, the positive results were specifically yielded with the genomic DNA from *H. influenzae*, while the negative results were detected with *H. parainfluenzae*, *H. haemolyticus*, *H. parahaemolyticus* and non-*H. influenzae* strains. All results indicated that the specificity of *H. influenzae* LAMP-LFB assay by colorimetric indicator (Fig. 5A), agarose gel electrophoresis (Fig. 5B) were conformity with LFB method (Fig. 5C), which has 100% specificity for *H. influenzae* detection.

### Examination of LAMP-LFB assay for clinical samples

**Fig. 6:**
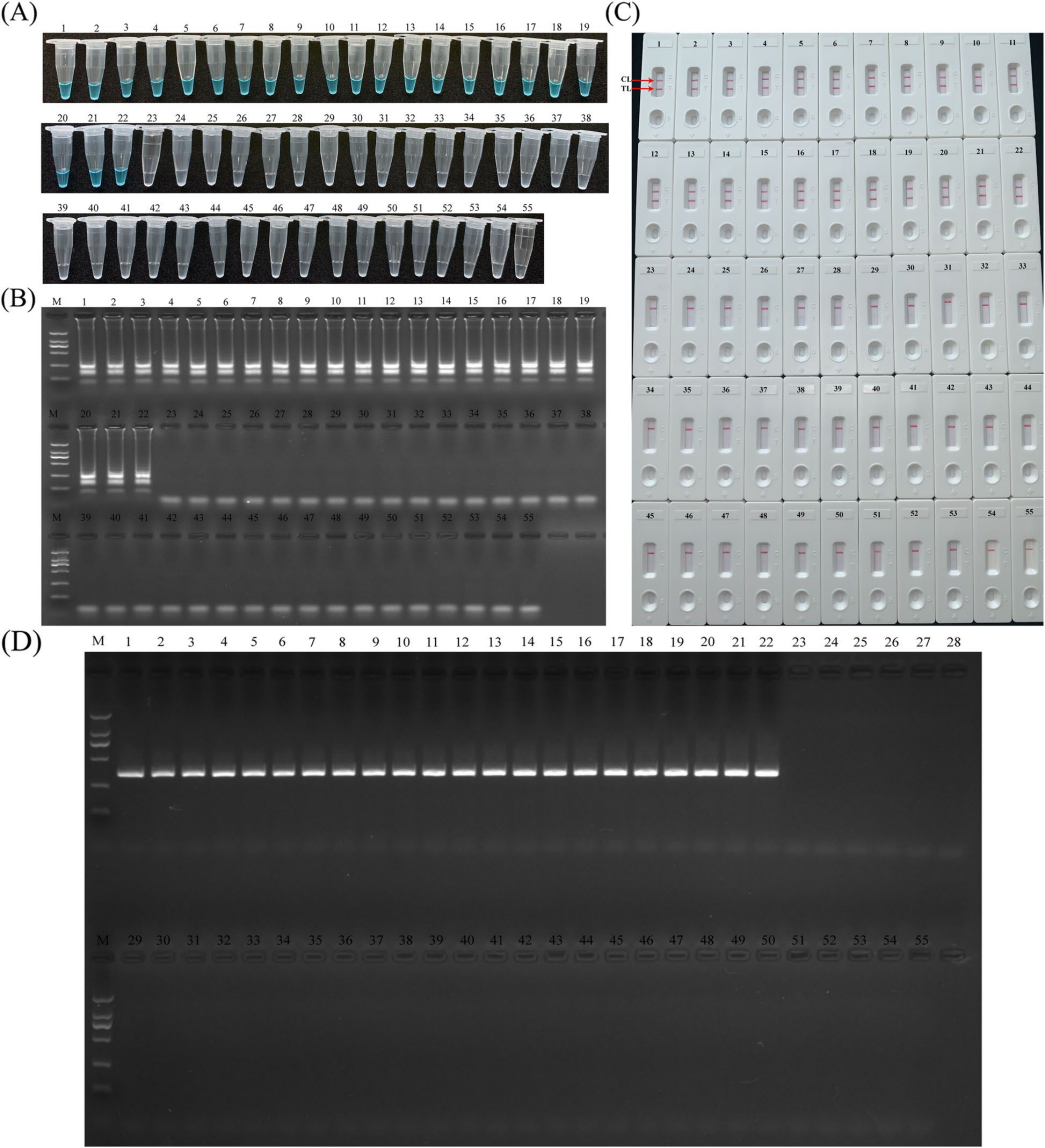
Detection of *H. influenzae* in clinical samples with LAMP-LFB assay and PCR. **A** The visible color change of *H. influenzae*-LAMP assay was demonstrated by colorimetric indicators; **B** Amplification products of *H. influenzae*-LAMP were detected by agarose gel electrophoresis; **C** The products of *H. influenzae*-LAMP were detected with LFB. **D** PCR method for detection of *H. influenzae.* The number from 1 to 22 represented the positive results. Other numbers showed the negative results

As a detection tool, the usability of LAMP-LFB method for diagnosing *H. influenzae* was evaluated with 55 DNA temples extracted from sputum samples. 5 μl DNA template from each sample was applied to *H. influenzae*-LAMP assay, each reaction was repeated three times, then the 0.5 μl reaction products were detected by LFB. The 22 of 55 sputum samples exhibited *H. influenzae* positive results in colorimetric indicator (Fig. 6A), agarose gel electrophoresis (Fig. 6B), and LFB analysis (Fig. 6C), which was completely in consistent with traditional cultivation detection results and PCR results (Fig. 6D).

## Discussion

As an exclusively human pathogen*, H. influenzae* is well recognized to be an important cause of respiratory infection and a major cause of systemic diseases such as community-acquired pneumonia, meningitis, bacteremia and otitis media in young children and infants, which need medical emergency requiring immediate diagnosis and treatment [1]. Unfortunately, from isolation to identification of *H. influenzae*, the traditional diagnose strategy was spending too much time and expensive. In our study, we adopted a convenient LAMP-LFB detection system to diagnose target pathogen. Obviously, only *H. influenzae* strains showed the positive results for LAMP-LFB compared with the control strains, such as *H. parainfluenzae*, *H. haemolyticus*, *H. parahaemolyticus* and non-*H. influenzae* strains*.* Thus, LAMP-LFB assay showed no cross-reactions to the above negative strains, which demonstrated that the specificity of the target pathogen detection arrived at 100%.

The sensitivity analysis showed that the minimum content of the target strain is 100 fg in *H. influenzae*-LAMP-LFB assay using the OMP *P6* gene. Meanwhile, within 52 min, the whole experimental steps of LAMP-LFB could be completed, which included 20 min for genomic template preparation, LAMP reaction time of 30 min (Fig. 4), and LFB analysis of 2 min. Compared with agarose gel electrophoresis, colorimetric indicator and real-time turbidity, the LFB method not only presented the reliable sensitivity and accuracy (Fig. 3), but also simpler and faster. For further evaluation the practicality of LAMP-LFB method to target pathogens, we detected 55 clinical sputum samples using biological culture method and LAMP-LFB detection, respectively. The LAMP-LFB technique revealed high specificity for *H. influenzae* strains in the sputum samples, which was in accordance with culture-biotechnical assay. In comparison with PCR and culture assays, *H. influenzae-*LAMP-LFB technique just need a thermostatic instrument with a constant temperature of 65℃, which effectively avoided the long turnaround times, expensive instruments, thermal denature and change in reaction temperature, suggesting the LAMP-LFB assay was an alternative to PCR-based method. Moreover, Syafirah et al. [27] found that the LAMP assay was at least 100-fold more sensitive than the PCR method for detection of *Vibrio cholerae.* Furthermore, recent research in *Mycoplasma pneumoniae* detection and *Staphylococcus aureus* detection showed that the LAMP-LFB method has better detection ability than the PCR method [22, 25].

The real-time turbidimeter, gel electropheresis, visual detection reagent (VDR) are all detection methods for LAMP amplification products. Each of those methods has its own advantages and disadvantages. Real-time turbidity method can monitor the reaction in real time, but it needs to rely on turbidimeter. Gel electropheresis, which requires an additional gel electrophoresis process of approximately 30 min. VDR method, although the naked eye can directly identify the reaction results, some negative reaction products also show a slight blue color, which may affect the judgment of the results. Herein, the LFB was employed to analyze LAMP products in *H. influenzae*-LAMP-LFB assay. The LFB relied on the primers of FIP labled with FITC and LF labled with biotin. And, the LFB method can complete the detection of amplified products within 5 min, and the results can be directly observed by naked eyes. Therefore, LAMP combined with LFB method was established in this study to detect *H. influenzae*. In compared with gel electrophoresis, colorimetric indicators and turbidity which applied in many previous reports, the LFB showed the superiority in ease of use in basic and clinical laboratories, simple operation and rapid results after the amplification process was completed. Moreover, based on the LFB, the extra procedure, special reagents, complicated instruments are all no longer needed. Furthermore, the results indicated with LFB is less subjective. Nevertheless, the LAMP-LFB detection also has limitation, since the LAMP results are shown qualitatively by red strips.

## Conclusion

The LAMP-LFB assay targeted the specific OMP *P6* gene of *H. influenzae* was successfully developed. The assay showed high selectivity for *H. influenzae* detection, high sensitivity of 100 fg in per reaction with pure culture. Meanwhile, the protocol is much more convenient with less time-spending and no expensive equipment. *H. influenzae*-LAMP-LFB assay established in this study might be used as a diagnosis tool for target pathogens, which would allow clinicians to make better informed decisions regarding patient treatment without delay.

## Materials and methods

### Instruments and reagents

The Loopamp kits and visual detection reagent (VDR) [22, 25] were purchased from HaiTaiZhengYuan Technology Co., Ltd (Beijing, China). The VDR has been widely used, and the VDR reagent is the obvious color contrast before and after reaction. Before the reaction, the VDR is light blue. In the positive reaction, the VDR continues to remain light blue. While, in the negative reaction, the VDR becomes colorless [22, 25]. The LFB, involving backing card, absorbent pad, conjugate pad, sample pad, nitrocellulose membrane (NC), and the isothermal amplification kit were purchased from Jie-Yi Biotechnology. Co., Ltd. (Shanghai, China). The *Bst* DNA Polymerase large Fragment was purchased from New England Biolabs Co., Ltd. (Beijing, China). The crimson red dye streptavidin-coated polymer nanoparticles (10 mg/mL, 100 mM borate, 0.05% Tween-20 with 10 mM EDTA, pH 8.5 with 0.1% BSA, 129 nm) were purchased from Bangs Laboratories, Inc. (India, USA). The rabbit anti-fluorescein antibody and the biotinylated bovine serum albumin were purchased from Abcam. Co., Ltd. (Shanghai, China). The FastPure® Blood/Cell/Tissue/Bacteria DNA Isolation Mini Kit was purchased from Vazyme biotech co., Ltd (Nanjing, China). The LA-320C realtime turbidimeter was purchased from Eiken Chemical Co., Ltd (Tokyo, Japan). The LA-320C realtime turbidimeter belongs to the special gene amplification assay device for LAMP method. The device can complete the entire process from gene amplification to detection. After incubation under isothermal conditions (60–65℃), the presence of target genes was determined by measuring the turbidity of magnesium pyrophosphate, a by-product of the amplified genes. The turbidity of each sample is measured in real time and the results are graphically displayed on the computer. The turbidity of each sample was measured every 6 s. The measurement is transferred to a computer to confirm the extent of amplification.

### Design of the LAMP primers

**Fig. 7:**
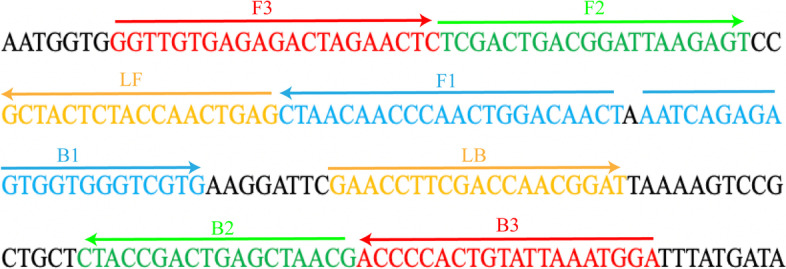
The LAMP primers designed according to OMP *P6* gene. Different colors were used to lable the 8 primers. The sense and anti-sense sequences were marked by right and left arrows, respectively

According to the *H. influenzae* OMP *P6* gene (Genbank accession no. L42023)*.* The specific LAMP primers listed in Table 1, involving F3, B3, BIP, FIP, LF and LB, were designed in terms of the reaction mechanism of LAMP-LFB method with PrimerExplorer V4 (Eiken Chemical) [28, 29]. Moreover, the FIP labeled with FITC at 5’end and LF labeled with biotin at 5’end were also listed in Table 1. Through the BLAST analysis, the specificity of LAMP primers was verified. The sequences and locations of primers were displayed in Fig. 7. All of the primers were synthesized by TSINGKE Biological Technology Co., Ltd. (Beijing, China) at HPLC purification grade.

**Table 1:** The primers used in this study

Primers	Sequences and modifications (5’-3’)	Gene
F3	GGTTGTGAGAGACTAGAACTC	OMP *P6*
B3	TCCATTTAATACAGTGGGGT	
FIP	AGTTGTCCAGTTGGGTTGTTAG -TCGACTGACGGATTAAGAGT	
FIP*	5’-FITC-AGTTGTCCAGTTGGGTTGTTAG -TCGACTGACGGATTAAGAGT-3’	
BIP	AATCAGAGAGTGGTGGGTCGTG-CGTT AGCTCAGTCGGTAG	
LF	CTCAGTTGGTAGAGTAGC	
LF*	5’-Biotin-CTCAGTTGGTAGAGTAGC-3’	
LB	GAACCTTCGACCAACGGAT	

^FIP*, 5’^^−^^labeled with FITC; FITC, fluorescein isothiocyanate; LF*, 5’^^−^^labeled with Biotin^

### Bacterial strains and genomic DNA preparation

The 39 clinical strains were detected in our study, including 10 *H. influenzae* strains, 3 *H. parainfluenzae*, 3 *Haemophilus haemolyticus*, 3 *Haemophilus parahaemolyticus* and 20 non-*H. influenzae* strains (Table 2). According to the instructions of FastPure® Blood/Cell/Tissue/Bacteria DNA Isolation Mini Kit (Nanjing, China), the genomic DNA of 39 strains were extracted. The extraction steps were as follows: For Gram-negative bacteria samples, the 200 μl Buffer ACL, 20 μl Proteinase K, and 200 μl Buffer BCL were added in turn. After Vortex blending, the mixtures were in 56℃ water bath for 10 min. For Gram-positive bacteria samples, 180 μl Lysozyme were added. After 37℃ water bath for 30 min, 20 μl Proteinase K, and 200 μl Buffer BCL were added in turn. After Vortex blending, the mixtures were in 56℃ water bath for 10 min. Next, 150 μl anhydrous ethanol was added and mixed. Transferring the mixture to the adsorption column for genome adsorption, 12,000 rpm 1 min. 500 μl Buffer WA were added for removing impurities such as protein, 12,000 rpm 1 min. 600 μl Buffer WB were added for removing salt ions, 12,000 rpm 1 min. Then, for removing ethanol, the adsorption columns were centrifuged at 12,000 rpm for 2 min and standed at room temperature for 5 min. Finally, the adsorption columns were added 50 μl Elution Buffer and standed 5 min. Subsequently, 12,000 rpm 1 min for genome elution. After extraction, with ultraviolet spectrophotometer (NanoDrop One, Thermo, America), the templates were measured at A260/280, and stored under at -20℃ until they were used. The reference strain was set with an isolate of *H. influenzae*, which was used in the sensitivity and optimisation analysis with pure culture. The 55 sputum samples for exhibiting the feasibility of LAMP-LFB in clinical detection were acquired from patients of Dingzhou People’s hospital with the written informed consent. This study was approved by the Ethics Committee of Dingzhou people’s Hospital, and conducted according to the medical research regulations of the Ministry of Health, China. The patients who were suspected of having *H. influenzae*, and sputum samples were collected over a period of three straight months.

**Table 2:** Bacterial strains used in this study

Bacteria species	Isolates (source)	No. of strains
*Haemophilus influenzae*	Isolates	10
*Haemophilus parainfluenzae*	Isolates	3
*Haemophilus haemolyticus*	Isolates	3
*Haemophilus parahaemolyticus*	Isolates	3
*Citrobacter braakii*	Isolates	2
*Streptococcus agalactiae*	Isolates	1
*Staphylococcus haemophilus*	Isolates	1
*Proteus mirabilis*	Isolates	1
*Streptococcus suis*	Isolates	2
*Listeria monocytogenes*	Isolates	2
*Listeria innocua*	Isolates	1
*Escherichia Coli*	Isolates	2
*Kleber pneumoniae*	Isolates	3
*Escherichia Coli*	Isolates	5

### Preparation of lateral flow biosensor

According to previous reports [23, 30, 31], the results of LAMP were detected by using LFB in this study. The LFB is a commercial kit were purchased from Jie-Yi Biotechnology. Co., Ltd. (Shanghai, China). Briefly, a conjugate pad, a absorbent pad, an immersion pad, nitrocellulose membrane and a backing pad were involved in LFB. The dye streptavidin-coated polymer nanoparticles were got together in the conjugated pad. According to the instructions, 0.5 ul LAMP amplification products were added on the LFB pad, the final result will be realistic in 5 min. After that, biotin-BSA and anti-FITC were restrained at test line (TL) and control line (CL).

### The standard LAMP-LFB assay

The standard LAMP reaction was conducted in a mixture of 25 μl based on previous research [32]. The reaction system was 25 μl including 2 × reaction mix 12.5 μl, DNA template 5 μl, FIP* primer 0.8 μM, FIP primer 0.8 μM, LF* primer 0.4 μM, LF primer 0.4 μM, F3 primer 0.4 μM, B3 primer 0.4 μM, BIP primer 1.6 μM, LB primer 0.8 μM, and 8 U *Bst* DNA polymerase large Fragment 1 μl. The amplification reaction was firstly conducted at 63℃ for 1 h. Afterwards, the reaction terminated at 85℃ for 5 min. The reaction system with 1 μl genomic template of *H. parainfluenzae*, *H. haemolyticus*, and *Staphylococcus aureus* strain were chosen as negative controls, respectively. In addition, the blank control reaction system contained 1 μl double distilled water. The detection methods, such as colorimetric indicators, agarose gel electrophoresis and LFB analysis, were applied to detect the LAMP amplification. According to the LFB instruction, 0.5 μl LAMP amplification product was dropped in the sample tank of LFB, then 3 drops of buffer were added to promote product diffusion. About 3–5 min later, the reaction results could be read.

### The optimal temperature for LAMP-LFB assay

To evaluate the optimal amplification temperature of *H. influenzae* LAMP-LFB assay*,* the amplifications were proceeded for 1 h and in the temperature range of 60℃ to 67℃ with 1℃ intervals. The real-time turbidimeter was used to monitored the reactions. Within 1 h and the threshold value > 0.1 were defined as positive reaction with 10 pg *H. influenzae* genomic DNA. While, the blank control contained 5 μl of distilled water. Each reaction performed three times.

### Analytical sensitivity of the LAMP-LFB assay

For verifying the limit of detection (LOD), the sensitivity of LAMP-LFB assays was proceeded with the gradient genomic DNA extracted from pure culture of *H. influenzae*, including 10 ng, 1 ng, 100 pg, 10 pg, 1 pg, 100 fg, 10 fg, and 1 fg. Four determination techniques, including real-time turbidimeter, colorimetric indicators, LFB analysis and agarose gel electrophoresis, were applied to detect the LAMP amplification. Each reaction conducted three times.

### The optimal reaction time of LAMP-LFB assay

The effect of LAMP amplification were examined at different times, and the reaction time was set from 10 to 60 min with 10 min interval, which conducted three times. The LAMP product was detected with colorimetric indicators and LFB.

### Specificity evaluation of the LAMP-LFB assay

In order to examine the specificity of the LAMP-LFB, the LAMP reactions were performed with the genomic templates (at least 10 ng/μl) from 10 *H. influenzae*, 3 *H. parainfluenzae*, 3 *H. haemolyticus*, 3 *H. parahaemolyticus* and 20 non-*H. influenzae* strains (Table 2). The colorimetric indicators, agarose gel electrophoresis and LFB analysis, were applied to detect the LAMP product. Each sample was analyzed three times independently.

## LAMP-LFB practical application in clinical samples

The 55 sputum samples mentioned in Bacterial Strains and Genomic DNA Preparation part were detected according to traditional culture methods, biochemical identification, colony morphology and Gram stain. As a result, *H. influenzae* isolates were successfully detected from 22 sputum samples. With DNA Isolation Kit as previously described in Reagents and Instruments part, the genomic DNA were extracted from 22 sputum samples that were positive for *H. influenzae* and another 33 randomly selected sputum samples that were negative for *H. influenzae*. The PCR carried out according to Torigoe [1] and LAMP method were used to detect *H. influenzae* in all of the 55 DNA temples. The colorimetric indicators, agarose gel electrophoresis and LFB analysis were used to analyze the LAMP amplification. Then the LAMP-LFB results and PCR results were compared to traditional culture.

## Supplementary Information

**Additional file 1.**

## Data Availability

The datasets generated and analysed during the current study are available in the GenBank repository. (Genbank accession no. L42023).

## References

[1] Torigoe H, Seki M, Yamashita Y, Sugaya A, Maeno M (2007). Detection of *Haemophilus influenzae* by loop-mediated isothermal amplification (LAMP) of the outer membrane protein P6 gene. Jpn J Infect Dis.

[2] Falla TJ, Crook DWM, Anderson EC, Ward JI, Santosham M, Eskola J, Moxon ER (1995). Characterization of capsular genes in Haemophilus influenzae isolates from H. influenzae type b vaccine recipients. J Infect Dis.

[3] Kroll JS, Ely S, Moxon ER (1991). Capsular typing of *Haemophilus influenzae* with a DNA probe. Mol Cell Probes.

[4] LaClaire LL, Tondella M, Beall DS, Noble CA, Raghunathan PL, Rosenstein NE, Popovic T (2003). Identification of *Haemophilus influenzae* serotypes by standard slide agglutination serotyping and PCR-based capsule typing. J Clin Microbiol.

[5] Muhlemann K, Balz M, Aebi S, Schopfer K (1996). Molecular characteristics of *Haemophilus influenzae* causing invasive disease during the period of vaccination in Switzerland: analysis of strains isolated between 1986 and 1993. J Clin Microbiol.

[6] van Ketel RJ, Wever BD, Alphen LV (1990). Detection of *Haemophilus influenzae* in cerebrospinal fluids by polymerase chain reaction DNA amplification. J Med Microbiol.

[7] Hendolin PH, Markkanen A, Ylikoski J, Wahlfors JJ (1997). Use of multiplex PCR for simultaneous detection of four bacterial species in middle ear effusions. J Clin Microbiol.

[8] Lu JJ, Perng CL, Lee SY, Wan CC (2000). Use of PCR with universal primers and restriction endonuclease digestions for detection and identification of common bacterial pathogens in cerebrospinal fluid. J Clin Microbiol.

[9] Murphy TF, Bartos LC, Campagnari AA, Nelson MB, Apicella MA (1986). Antigenic characterization of the P6 protein of nontypable *Haemophilus influenzae*. Infect Immun.

[10] Nelson MB, Munson RS, Apicella MA, Sikkema DJ, Molleston JP, Murphy TF (1991). Molecular conservation of the P6 outer membrane protein among strains of *Haemophilus influenzae*: analysis of antigenic determinants, gene sequences, and restriction fragment length polymorphisms. Infect Immun.

[11] Karalus RJ, Murphy TF (1999). Purification and characterization of outer membrane protein P6, a vaccine antigen of non-typeable *Haemophilus influenzae*. FEMS Immunol Med Mic.

[12] Notomi T, Okayama H, Masubuchi H, Yonekawa T, Watanabe K, Amino N, Hase T (2000). Loop-mediated isothermal amplification of DNA. Nucleic Acids Res.

[13] Mori Y, Kanda H, Notomi T (2013). Loop-mediated isothermal amplification (LAMP): recent progress in research and development. J Infect Chemother.

[14] Prusty BR, Chaudhuri P, Chaturvedi VK, Saini M, Mishra BP, Gupta PK (2016). Visual Detection of Brucella spp. in Spiked Bovine Semen Using Loop-Mediated Isothermal Amplification (LAMP) Assay. Indian J Microbiol.

[15] Pérez-Sancho M, García-Seco T, Arrogante L, Garcia N, Martinez I, Diez-Guerrier A, Perales A, Goyache J, Dominguez L, Alvarez J (2013). Development and evaluation of an IS711-based loop mediated isothermal amplification method (LAMP) for detection of Brucella spp. on clinical samples. Res Vet Sci.

[16] Soleimani M, Shams S, Majidzadeh-A K (2013). Developing a real-time quantitative loop-mediated isothermal amplification assay as a rapid and accurate method for detection of *Brucellosis*. J Appl Microbiol.

[17] Francois P, Tangomo M, Hibbs J, Bonetti EJ, Boehme CC, Notomi T, Perkins MD, Schrenzel J (2011). Robustness of a loop-mediated isothermal amplification reaction for diagnostic applications. FEMS Immunol Med Mic.

[18] Mori Y, Notomi T (2009). Loop-mediated isothermal amplification (LAMP): a rapid, accurate, and cost-effective diagnostic method for infectious diseases. J Infect Chemother.

[19] Kim DW, Kilgore PE, Kim EJ, Kim SA, Anh DD, Seki M (2011). Loop-mediated isothermal amplification assay for detection of *Haemophilus influenzae* type b in cerebrospinal fluid. J Clin Microbiol.

[20] Takano C, Seki M, Kim DW, Kilgore PE, Fuwa K, Takahashi K, Inazaki T, Hayakawa S (2017). Molecular serotype-specific identification of non-type b *Haemophilus influenzae* by loop-mediated isothermal amplification. Front Microbiol.

[21] Zhang X, Lowe SB, Gooding JJ (2014). Brief review of monitoring methods for loop-mediated isothermal amplification (LAMP). Biosens Bioelectron.

[22] Jiang LX, Li XM, Gu RM, Mu DG (2020). Nanoparticles-Based biosensor coupled with multiplex loop-mediated isothermal amplification for detection of Staphylococcus aureus and identification of methicillin-resistant S. aureus. Infect Drug Resist..

[23] Li S, Liu Y, Wang Y, Chen H, Liu C, Wang Y (2019). Lateral flow biosensor combined with loop-mediated isothermal amplification for simple, rapid, sensitive, and reliable detection of *Brucella spp*. Infect Drug Resist.

[24] Quesada-González D, Merkoci A (2015). Nanoparticle-based lateral flow biosensors. Biosens Bioelectron.

[25] Wang YC, Wang Y, Jiao WW, Li JQ, Quan ST, Sun L, Wang YH, Qi X, Wang XY, Shen A (2019). Development of loop-mediated isothermal amplification coupled with nanoparticle-based lateral flow biosensor assay for *Mycoplasma pneumoniae* detection. AMB Express.

[26] Zhu X, Wang X, Han L, Chen T, Wang LC, Li H, Li S, He LF, Fu XY, Chen SJ, Xing M, Chen H, Wang Y (2020). Multiplex reverse transcription loop-mediated isothermal amplification combined with nanoparticle-based lateral flow biosensor for the diagnosis of COVID-19. Biosens Bioelectron.

[27] Syafirah EAREN, Najian ABN, Foo PC, Ali MRM, Mohamed M, Yean CY (2018). An ambient temperature stable and ready-to-use loop-mediated isothermal amplification assay for detection of toxigenic *Vibrio cholerae* in outbreak settings. Acta tropica.

[28] Brakstad OG, Aasbakk K, Maeland JA (1992). Detection of Staphylococcus aureus by polymerase chain reaction amplification of the *nuc* gene. J Clin Microbiol.

[29] Liu D, Wang C, Swiatlo EJ, Lawrence ML (2005). PCR amplification of a species-specific putative transcriptional regulator gene reveals the identity of *Enterococcus faecalis*. Res microbiol.

[30] Wang Y, Liu D, Deng J, Wang Y, Xu J, Ye C (2017). Loop-mediated isothermal amplification using self-avoiding molecular recognition systems and antarctic thermal sensitive uracil-DNA-glycosylase for detection of nucleic acid with prevention of carryover contamination. Anal Chim Acta.

[31] Wang Y, Wang Y, Li D, Xu J, Ye C (2018). Detection of nucleic acids and elimination of carryover contamination by using loop-mediated iso-thermal amplification and antarctic thermal sensitive uracil-DNA-glycosylase in a lateral flow biosensor: application to the detection of *Streptococcus pneumoniae*. Microchim Acta.

[32] Wang Y, Wang Y, Lan R, Xu H, Ma A, Li D, Dai H, Yuan X, Xu J, Ye C (2015). Multiple Endonuclease Restriction Real-Time Loop-Mediated Isothermal Amplification: A Novel Analytically Rapid, Sensitive, Multiplex Loop-Mediated Isothermal Amplification Detection Technique. J mol diagn.

