# Supplementary material for: Detection of Haemophilus influenzae by loop-mediated isothermal amplification coupled with nanoparticle-based lateral flow biosensor assay
Source: BMC Microbiol. 2022 May 5;22:123. doi: 10.1186/s12866-022-02547-5 (PMC9069426; doi:10.1186/s12866-022-02547-5)
Supplement: Supplementary file 1 — Additional file 1. [file 12866_2022_2547_MOESM1_ESM.pdf]

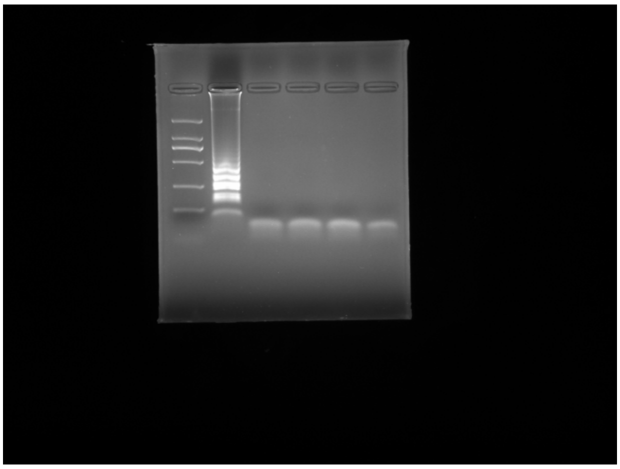

Original figure 2 Confirmation and demonstration of *H. influenzae*-LAMP amplification products. (B) Amplicon of *H. influenzae*-LAMP was analyzed by agarose gel electrophoresis

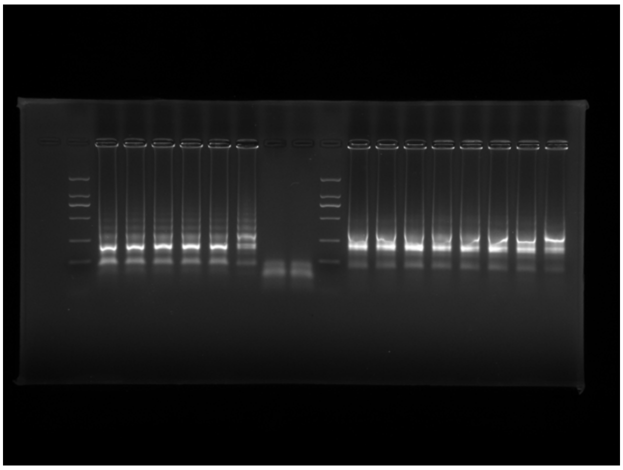

Original figure 4 Sensitivity of *H. influenzae* LAMP-LFB assay with gradient dilution of genomic DNA templates. (C) Agarose gel electrophoresis (the left part)

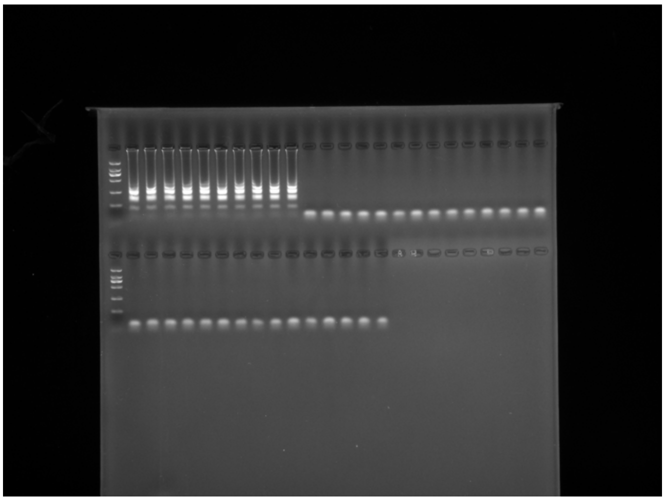

Original figure 6 Specificity of *H. influenzae* LAMP-LFB assay with different strains' DNA templates. (B) The LAMP products were detected with agarose gel electrophoresis

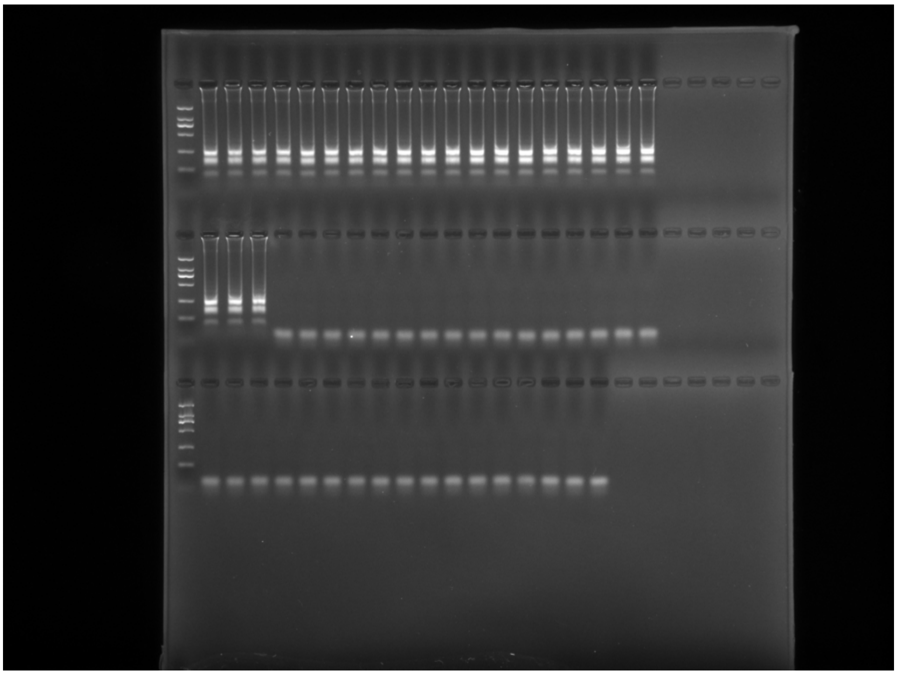

Original figure 7 Detection of *H. influenzae* in clinical samples with LAMP-LFB assay and PCR. (B) Amplification products of *H. influenzae*-LAMP were detected by agarose gel electrophoresis

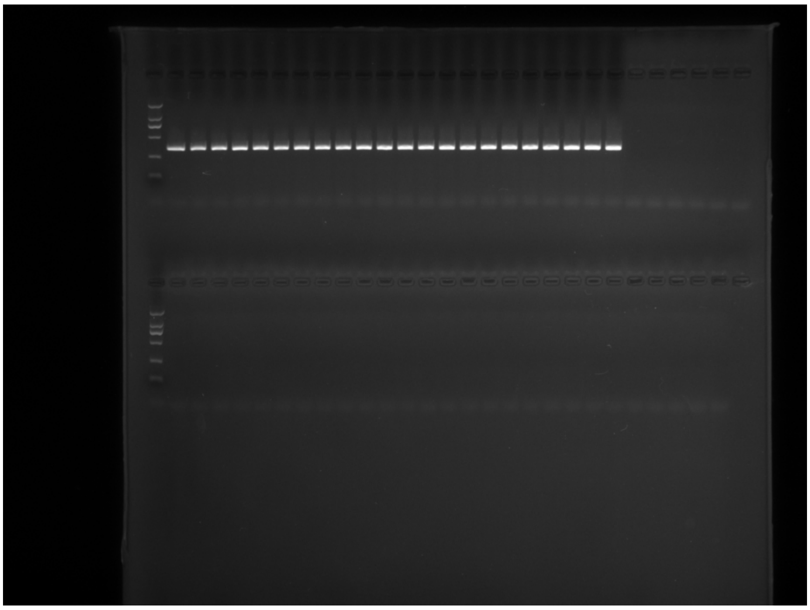

Original figure 7 Detection of *H. influenzae* in clinical samples with LAMP-LFB assay and PCR. (D) PCR method for detection of *H. influenzae*
